# Supplementary material for: Genome sequence of Ophryocystis elektroscirrha, an apicomplexan parasite of monarch butterflies: cryptic diversity and response to host-sequestered plant chemicals
Source: BMC Genomics. 2023 May 24;24:278. doi: 10.1186/s12864-023-09350-0 (PMC10210345; doi:10.1186/s12864-023-09350-0)
Supplement: Supplementary file 2 — Supplementary Material 2 [file 12864_2023_9350_MOESM2_ESM.docx]

**Genome size estimation with k-mers.**

Without independent priors for genome size (e.g. flow cytometry estimates or metrics from a closely related species), our best expectation for genome assembly comes from the raw sequence data themselves. Here we briefly explain the k-mer counting method of genome size estimation. The code for these analyses can be found at <http://www.github.com/amongue/OE_genome>. In short, a k-mer is a subset of a sequenced read of arbitrary length “k”, in our case k = 21. Each full sequenced read is composed of many k-mers, which is the advantage of splitting reads into k-mers. Whatever the error rate is for reads (e.g. 1:1000 for reads of Q30 quality), even an error read is likely to have only a single error. So, when splitting into component k-mers, most k-mers for an error containing read do not contain that error. Thus, error k-mers are easily distinguished by their rarity.


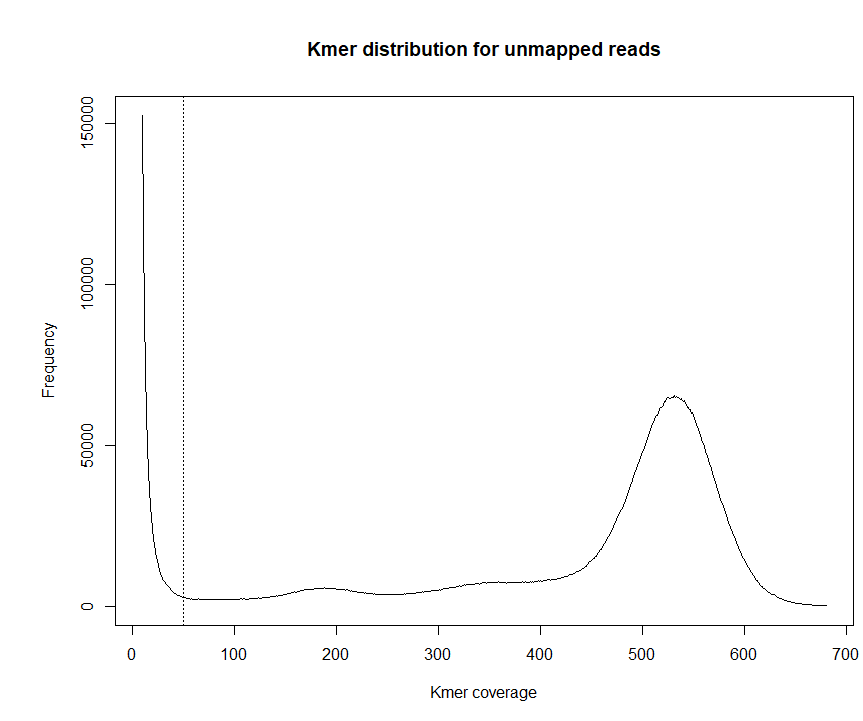

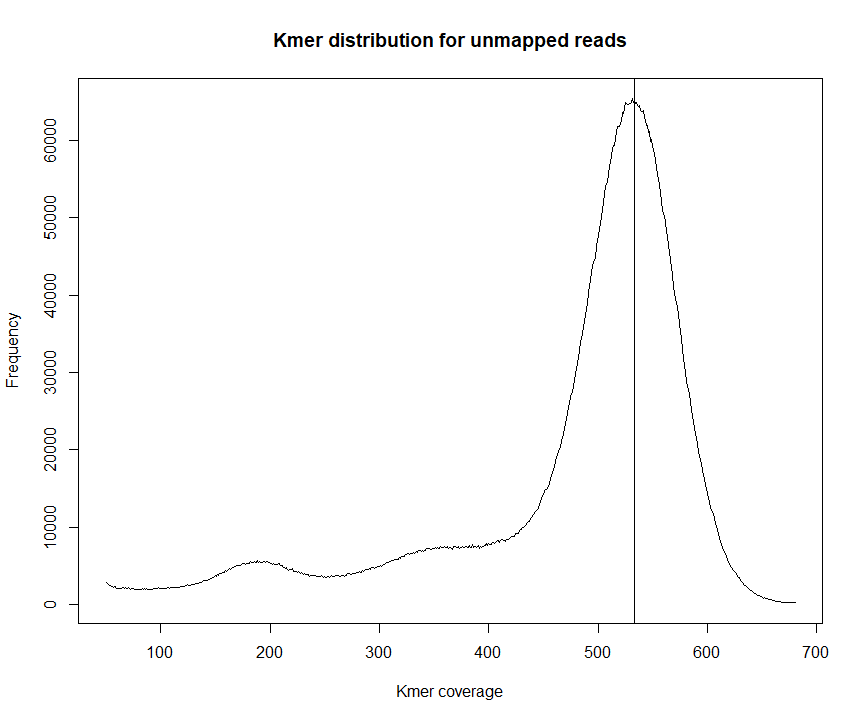


**Figure S1. K-mer plots used for genome size estimates. Left.** We plot the full distribution of 21-mers in our input for assembly of *O. elektroscirrha* (this analyses uses only unmapped reads from alignment to the host genome). The x-axis shows the coverage of a k-mer (how many times it is seen in the dataset). The y-axis is the summed frequency of all unique k-mers that have that coverage. A large spike of low frequency k-mers (<50x, left of the dotted line) represent likely sequencing errors. **Right.** We discard these errors from subsequent analyses, giving a clearer view of the maximum coverage (533x).

Under simple assumptions (e.g. ignoring repetitive sequences and ambiguous mapping), each k-mer is unique in the genome. The number of times a k-mer is seen should reflect the depth of sequencing coverage for the genome then (x-axis). Although sequencing coverage is never truly uniform, it follows a distribution centered on the modal coverage, 533x in our case. Finally, to calculate genome size, can take sum of all error-free k-mers (area under the curve from [50:700] above, and divide by our best estimate of coverage (533) to get the total length of a single copy of the genome represented in the sequenced reads. In the case of *O. elektroscirrha* we get 8,108,162 bases (see R script on github).


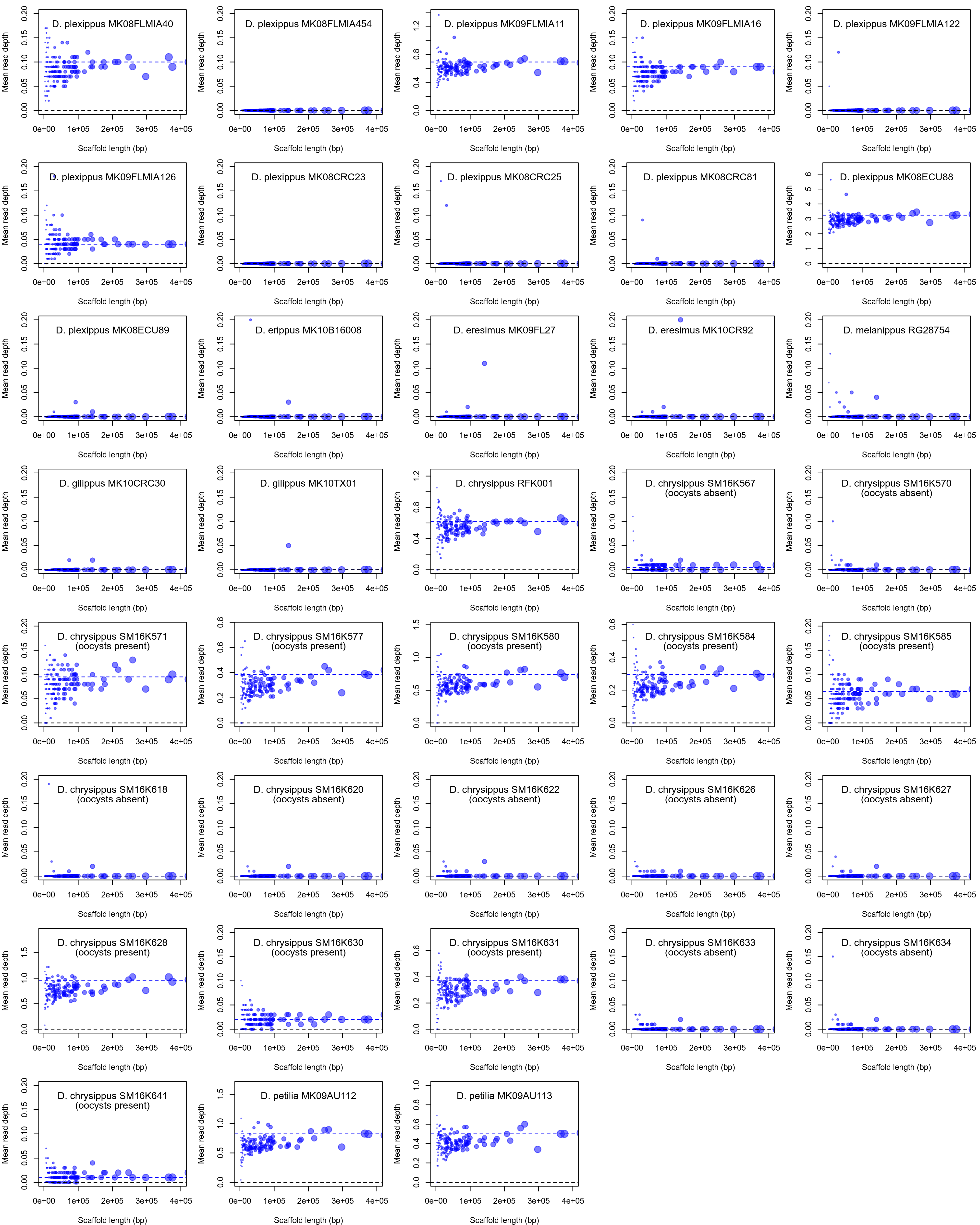


**Figure S2. Using genomic data from butterflies to diagnose *Ophryocystis* infection**

Mean read depth per scaffold in the *O. elektroscirrha* genome for Illumina sequencing reads generated from butterfly tissue is plotted against scaffold length. Each plot represents a separate butterfly sample, with species and ID indicated. Although short scaffolds show variable read depth, longer scaffolds can reliably detect infection in the form on non-zero average read length. Dashed horizontal lines indicate the median read depth for scaffolds >200kb in length. For 18 *D. chrysippus* samples, it is also indicated whether oocytes were detected on the sample. All nine positive cases correspond to cases where read depth is also non zero.


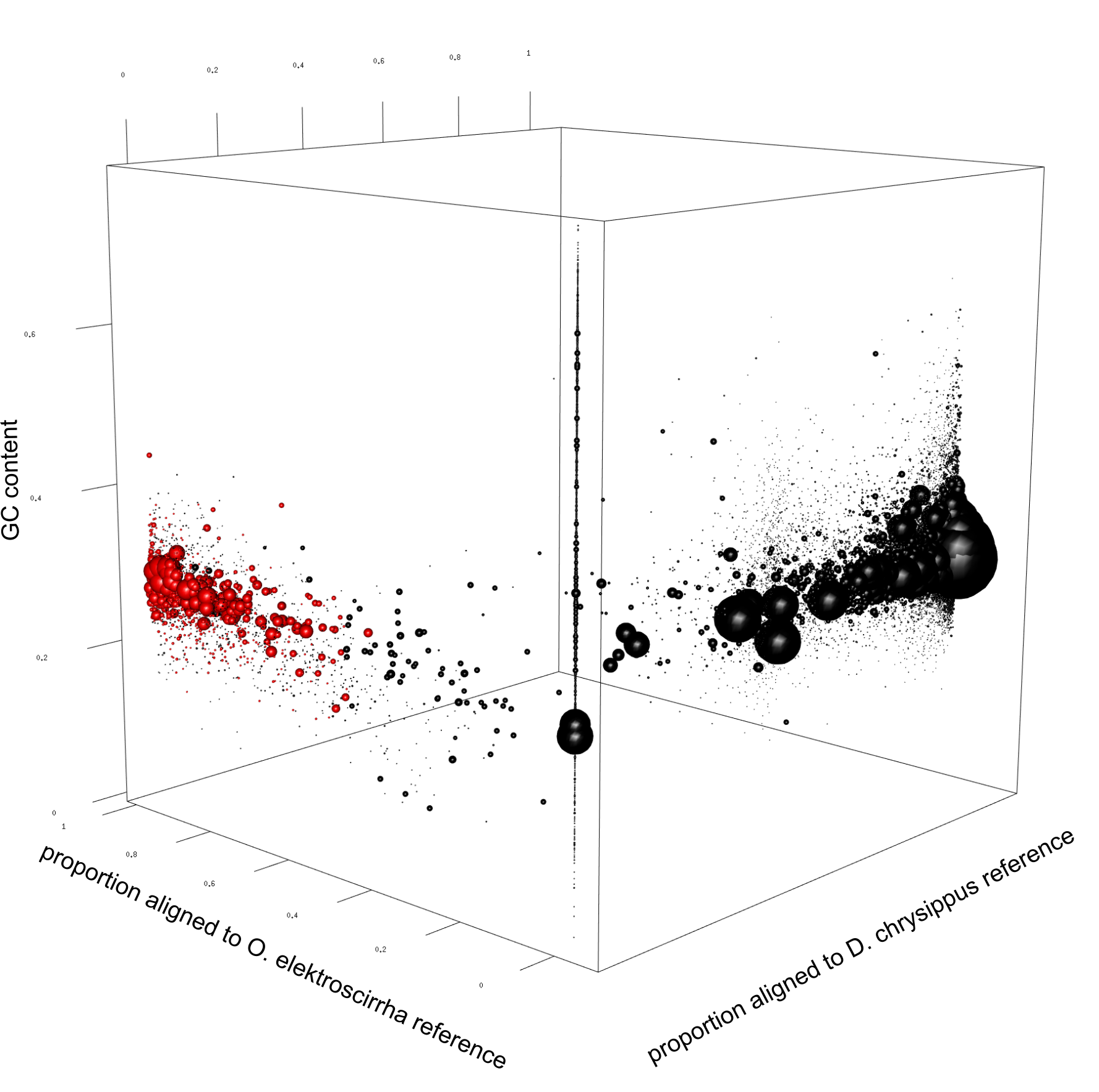


**Figure S3. Identification of *Ophryocystis*-like scaffolds in a *D. chrysippus* genome assembly**

Scaffolds identified as sufficiently *Ophryocystis*-like to represent a putative genome for an *Ophryocystis*-like parasite of *D. chrysippus* are indicated in red. GC content (vertical axis) is only slightly different on average between the host and parasite genomes, so this was not considered for identification of parasite scaffolds.


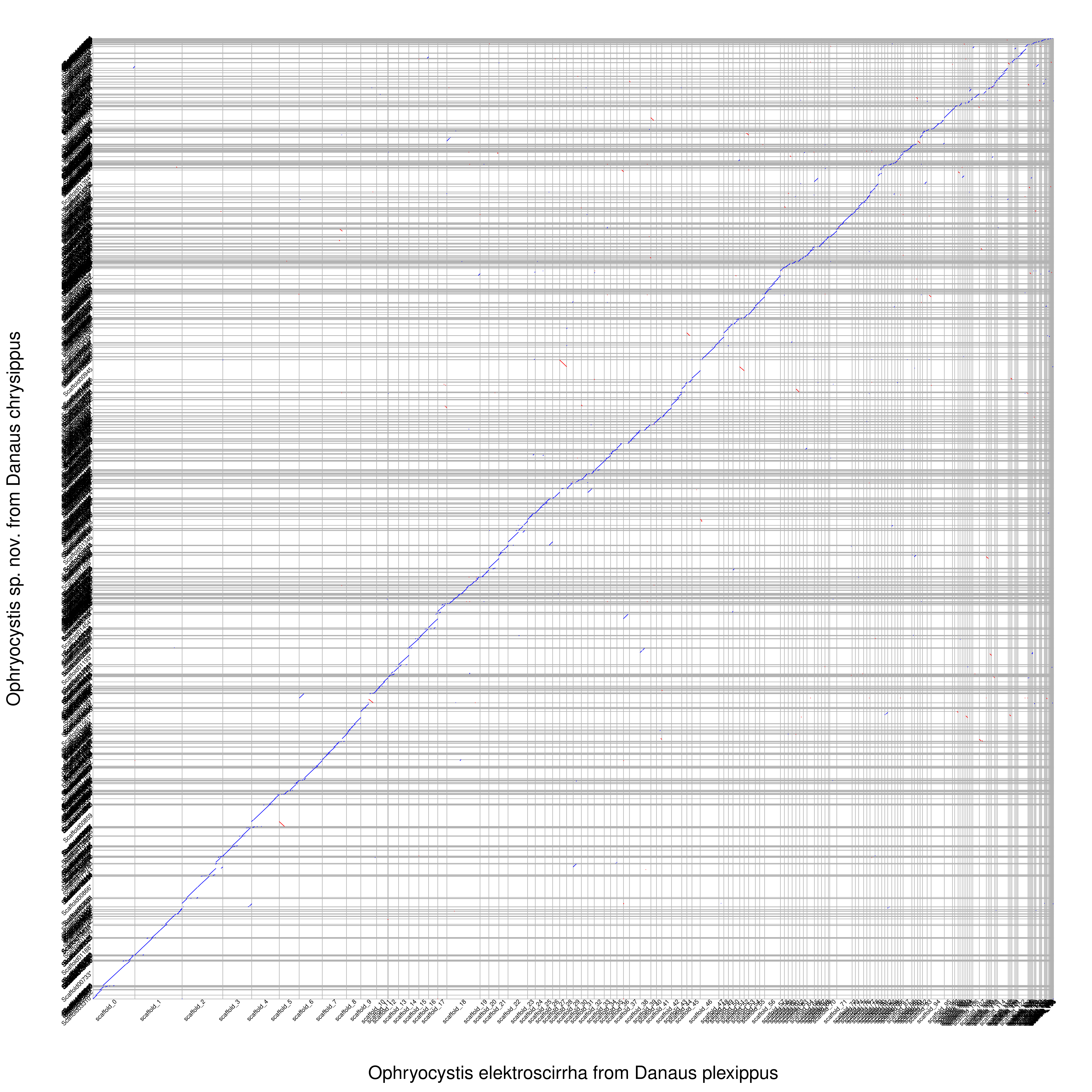


**Figure S4. Alignment between the genomes of O. *elektroscirrha* and the *Ophryocystis*-like parasite of *D. chrysippus***

Blue diagonal lines indicate tracts of aligned sequence between the two genomes. Scaffolds are separated by gray lines. The fact that most scaffolds have a complete and 1:1 alignment indicates that the genomes are likely both close to complete.

**Table S1. Orphaned orthologs.** In exploring conservation of BUSCO genes across *O. elektroscirrha*, *P. gigantea*, and *G. niphandrodes*, we found some genes in only 2 of the 3 lineages, and a smaller set of genes unique to only one of 3 lineages (Figure 2). Here we provide functional annotation information for these “orphaned orthologs” which may be of further interest to researchers as potentially lineage-specific features. Descriptions are based on OrthoDB. BUSCO IDs are given in parentheses for each description.

| *Ophryocystis elektroscirrha*  n = 7 | *Porospora gigantea A*  n = 6 | *Gregarina niphandrodes*  n = 19 | | |
| --- | --- | --- | --- | --- |
| Alkaline phosphatase  (26459at5794) | Eukaryotic initiation factor-2B gamma subunit  (27220at5794) | 26S Proteasome regulatory subunit 6A  (12351at5794) | Histidine kinase/HSP90-like ATPase  (2648at5794) | RNA-binding domain superfamily  (27360at5794) |
| Cap-specific mRNA (Nucleoside-2'-O-)-methyltransferase  (2281at5794) | Flavodoxin domain-containing protein  (7233at5794) | Autophagy-related protein 3  (28469at5794) | Methyltransferase domain protein  (20983at5794) | Signal peptidase  (28230at5794) |
| Eukaryotic translation initiation factor 3 subunit 5  (17497at5794) | Sec1-like protein  (6796at5794) | C2 Domain  (1911at5794) | Mitochondrial processing peptidase  (11284at5794) | Succinyl-CoA ligase alpha subunit  (14681at5794) |
| Mitochondrial 2-oxoglutarate/malate carrier protein  (19637at5794) | Mog1 protein, putative  (32865at5794) | DnaJ Domain  (15514at5794) | Nucleosome assembly protein  (25213at5794) | Transcription elongation factor 140 kDa  (3086at5794) |
| Mitochondrial import inner membrane translocase subunit tim17  (27293at5794) | Ubiquitin-conjugating enzyme  (27522at5794) | Emp24/gp25L/p24 family domain-containing transmembrane protein  (25859at5794) | Oxoglutarate dehydrogenase (Succinyl-transferring), E1 component  (2374at5794) | Ubiquitin-conjugating enzyme  (22709at5794) |
| C2 Domain  (1166at5794) | Erythrocyte membrane-associated antigen  (9319at5794) | Eukaryotic translation initiation factor 3 subunit G  (22116at5794) | P-type ATPase  (4152at5794) |  |
| Small nuclear ribonucleoprotein E  (31976at5794) |  | Heat shock protein 110  (5067at5794) | Poly(A) polymerase PAP  (8055at5794) |  |

**Table S2. Accessions and visual parasite infection validation for sequence-based infection screening.**

Included as a separate excel table are the identities of butterfly samples we bioinformatically screened for *Ophryocystis* infection. All samples are assigned an infection status based on this method (read alignment). For a subset, we also had access to the host tissues and visually confirmed infection via microscopy (oocysts on scales).
